# The translesion DNA polymerases Pol ζ and Rev1 are activated independently of PCNA ubiquitination upon UV radiation in mutants of DNA polymerase δ

**DOI:** 10.1371/journal.pgen.1007119

**Published:** 2017-12-27

**Authors:** Carine Tellier-Lebegue, Eléa Dizet, Emilie Ma, Xavier Veaute, Eric Coïc, Jean-Baptiste Charbonnier, Laurent Maloisel

**Affiliations:** 1 I2BC, CEA, CNRS, Univ. Paris-Sud, Univ. Paris-Saclay, Gif-sur-Yvette, France; 2 LRGM, iRCM, IBFJ, DRF, CEA-Université Paris-Saclay, Fontenay-aux-Roses, France; Duke University, UNITED STATES

## Abstract

Replicative DNA polymerases cannot insert efficiently nucleotides at sites of base lesions. This function is taken over by specialized translesion DNA synthesis (TLS) polymerases to allow DNA replication completion in the presence of DNA damage. In eukaryotes, Rad6- and Rad18-mediated PCNA ubiquitination at lysine 164 promotes recruitment of TLS polymerases, allowing cells to efficiently cope with DNA damage. However, several studies showed that TLS polymerases can be recruited also in the absence of PCNA ubiquitination. We hypothesized that the stability of the interactions between DNA polymerase δ (Pol δ) subunits and/or between Pol δ and PCNA at the primer/template junction is a crucial factor to determine the requirement of PCNA ubiquitination. To test this hypothesis, we used a structural mutant of Pol δ in which the interaction between Pol3 and Pol31 is inhibited. We found that in yeast, *rad18Δ-*associated UV hypersensitivity is suppressed by *pol3-ct*, a mutant allele of the *POL3* gene that encodes the catalytic subunit of replicative Pol δ. *pol3-ct* suppressor effect was specifically dependent on the Rev1 and Pol ζ TLS polymerases. This result strongly suggests that TLS polymerases could rely much less on PCNA ubiquitination when Pol δ interaction with PCNA is partially compromised by mutations. In agreement with this model, we found that the *pol3-FI* allele suppressed *rad18Δ-*associated UV sensitivity as observed for *pol3-ct*. This *POL3* allele carries mutations within a putative PCNA Interacting Peptide (PIP) motif. We then provided molecular and genetic evidence that this motif could contribute to Pol δ-PCNA interaction indirectly, although it is not a *bona fide* PIP. Overall, our results suggest that the primary role of PCNA ubiquitination is to allow TLS polymerases to outcompete Pol δ for PCNA access upon DNA damage.

## Author summary

Replicative DNA polymerases have the essential role of replicating genomic DNA during the S phase of each cell cycle. DNA replication occurs smoothly and accurately if the DNA to be replicated is undamaged. Conversely, replicative DNA polymerases stall abruptly when they encounter a damaged base on their template. In this case, alternative specialized DNA polymerases are recruited to insert nucleotides at sites of base lesions. However, these translesion polymerases are not processive and they are poorly accurate. Therefore, they need to be tightly regulated. This is achieved by the covalent binding of the small ubiquitin peptide to the polymerase cofactor PCNA that subsequently triggers the recruitment of translesion polymerases at sites of DNA damage. Yet, recruitment of translesion polymerases independently of PCNA ubiquitination also has been documented, although the underlying mechanism is not known. Moreover, this observation makes more difficult to understand the exact role of PCNA ubiquitination. Here, we present strong genetic evidence in *Saccharomyces cerevisiae* implying that the replicative DNA polymerase δ (Pol δ) prevents the recruitment of the translesion polymerases Pol ζ and Rev1 following UV irradiation unless PCNA is ubiquitinated. Thus, the primary role of PCNA ubiquitination would be to allow translesion polymerases to outcompete Pol δ upon DNA damage. In addition, our results led us to propose that translesion polymerases could be recruited independently of PCNA ubiquitination when Pol δ association with PCNA is challenged, for instance at difficult-to-replicate loci.

## Introduction

Despite the remarkable catalytic activities of replicative DNA polymerases, these enzymes cannot efficiently incorporate nucleotides opposite damaged template DNA. Therefore, in eukaryotes, translesion DNA synthesis (TLS) is carried out by specialized, low stringency damage-tolerant polymerases belonging to the Y-family (Pol η, Pol ι, Pol κ, and Rev1) and the B family (Pol ζ) [1]. The inevitable consequence of the TLS polymerases feature to synthesize across DNA lesions with no associated proofreading activity, is their overall reduced fidelity, even at undamaged templates. This could lead to accumulation of unwanted mutations and therefore, their activity needs to be tightly regulated [2,3].

The main mechanism of damage-induced activation of TLS polymerases involves covalent modifications of the sliding clamp PCNA by ubiquitin or SUMO peptides [4–7]. PCNA mono-ubiquitination at the highly conserved lysine (K) 164 by the ubiquitin-conjugating enzyme (E2) Rad6 and the ubiquitin ligase (E3) Rad18 is a prerequisite for the activation of TLS polymerases [4,8,9]. Subsequently, K164 is poly-ubiquitinated via a K63-linked poly-ubiquitin chain, for which Rad5 and the Mms2-Ubc13 complex are additionally required. PCNA poly-ubiquitination then triggers a template switch mechanism [4,10].

The recruitment of DNA replication and repair proteins to DNA by PCNA is often dependent on the presence of a conserved protein-protein interaction motif, the "PCNA-interacting protein" or PIP-box [11]. The PIP-box consensus sequence, **Q**xx(**M/L/I**)xx**F(Y/F)**, is well conserved [12–14]. All Y family DNA polymerases interact with PCNA. PIP domains are found in Pol η, Pol ι and Pol κ [15–21], whereas Rev1 interacts with PCNA through an additional motif [22]. In addition, one or two ubiquitin-binding domains (UBDs) were identified in all eukaryotic members of the Y family [23,24]. They are the prototypes of two distinct classes: the ubiquitin-binding zinc finger (UBZ) and the helical ubiquitin-binding motif (UBM). Mutational inactivation of these motifs abolishes TLS in yeast and prevents damage-induced association of the mutated polymerases with PCNA in mammalian cells [22–29].

*In vitro* biochemical assays showed that ubiquitinated PCNA activates the replicative Pol δ similarly to unmodified PCNA [30,31]. Pol η takes the place of Pol δ at the 3’ extending end only when DNA synthesis by Pol δ is stalled and PCNA is ubiquitinated [32,33]. These observations lend support to an expanded "tool-belt" model [2]: ubiquitin primarily acts as a supplementary interaction module to which TLS polymerases first bind before the switch between polymerases. Support for this model comes from structural studies on ubiquitinated PCNA, the catalytic core of Pol η, its PIP-box bound to PCNA and its UBZ domain in complex with ubiquitin [34–38]. Particularly, it was observed that ubiquitin attachment to PCNA does not alter its conformation. Moreover, the ubiquitin moiety is positioned on the back side of the PCNA ring, presumably far away from its front side where PIP binding sites are localized and where polymerases position themselves and perform DNA synthesis [37,38].

Although the role of PCNA ubiquitination is firmly established, a number of observations indicate that TLS polymerases can be recruited also in the absence of PCNA modification. In yeast, early genetic studies showed that spontaneous mutagenesis in wild type (WT) and in the *rad18Δ* mutant, in which PCNA cannot be ubiquitinated, depends on Pol ζ and Rev1 [39–41]. More recently, studies using human cell extracts [42,43], mouse embryo fibroblasts [44–46], mouse pre-B cells [45] or DT40 chicken cells [47,48] reported activation of TLS polymerases with unmodified PCNA in various contexts: bypass of site-specific DNA lesions on plasmids, somatic hypermutation on immunoglobulin genes, and UV radiation- or methyl methanesulfonate-induced DNA damage. These results are puzzling because the conditions that allow the recruitment of TLS polymerases independently of PCNA ubiquitination are currently unknown.

The finding that Pol δ and TLS polymerases share common structural features is another challenging issue to understand the preferential recruitment of Pol δ over TLS polymerases. Pol δ from *S*. *cerevisiae* has three subunits: Pol3, Pol31 and Pol32. Pol3 is the catalytic subunit that contains the polymerase and the 3’ to 5’ exonuclease active site domains. In addition, Pol3 carries a C-terminal domain (CTD) with eight conserved cysteine residues that is folded distinctly from the catalytic domain. The first set of four cysteines (CysA) resembles a zinc ribbon motif [49] and is crucial for mediating DNA-dependent interactions between PCNA and Pol δ [50]. The second C-terminal set of cysteines (CysB) is an essential Fe-S cluster [50]. Pol31 is the essential structural B subunit of 55 kDa with which Pol3 CTD interacts. Pol32 is a non-essential subunit of 40 kDa (or C subunit) that is tethered solely *via* interactions with Pol31 [51]. Moreover, similarly to some TLS polymerases, Pol δ carries PIP motifs. A canonical PIP motif lies in the C-terminal end of Pol32 and has been shown to interact with PCNA by two-hybrid analyses [52]. Non-canonical PIP motifs are present in Pol3 and Pol31 and they contribute to PCNA-stimulated DNA synthesis [53]. Thus, the presence of multiple distant PIP motifs on the different Pol δ subunits could provide a positive advantage for the access to PCNA compared with monomeric TLS polymerases that carry one or several adjacent PIP motifs [20,21].

More surprisingly, Pol δ and Pol ζ share the same B and C subunits (Pol31 and Pol32 in yeast; P50 and P66 in mammals) [54–56]. This raises the possibility that Pol δ preferential access to unmodified PCNA is determined mostly *via* its catalytic subunit. It has been proposed that in yeast, the Rev3/Rev7 catalytic complex of Pol ζ replaces Pol3 on the Pol31/Pol32 platform to allow the polymerase switch from Pol δ to Pol ζ upon DNA damage [54]. In this scenario, PCNA ubiquitination could trigger the specific poly-ubiquitination of Pol3 by Def1 and its subsequent proteosomal degradation [57].

Given these structural similarities between Pol δ and TLS polymerases, we hypothesized that destabilization of the interaction between Pol δ subunits and/or between Pol δ and PCNA could modify the regulation of replicative and TLS polymerase loading on PCNA. To this aim, we used a mutant allele of *POL3* (*pol3-ct*). First we showed that in budding yeast, this mutant leads to an increase of UV resistance in *rad18Δ* cells. We then demonstrated that the suppression of the *rad18Δ* phenotype by *pol3-ct* occurs *via* Pol ζ and Rev1, although PCNA is not ubiquitinated. Moreover, mutational inactivation of Pol3 non-canonical PIP motif in the *rad18Δ* mutant led also to a robust increase in UV resistance. This suggests that a partial loss of the Pol δ-PCNA interaction is responsible for the increased UV resistance, when PCNA is not ubiquitinated. Using isothermal titration calorimetry (ITC), we found that Pol3 non-canonical PIP motif is probably not a *bona fide* PIP domain and might participate only indirectly in the PCNA-Pol δ interaction. Overall, our results suggest that the stability of the interaction between PCNA and Pol δ a the primer/template junction is a crucial factor to determine the requirement of PCNA ubiquitination.

## Results

### *pol3-ct* suppresses UV hypersensitivity associated with *RAD18* inactivation

**Fig 1:**
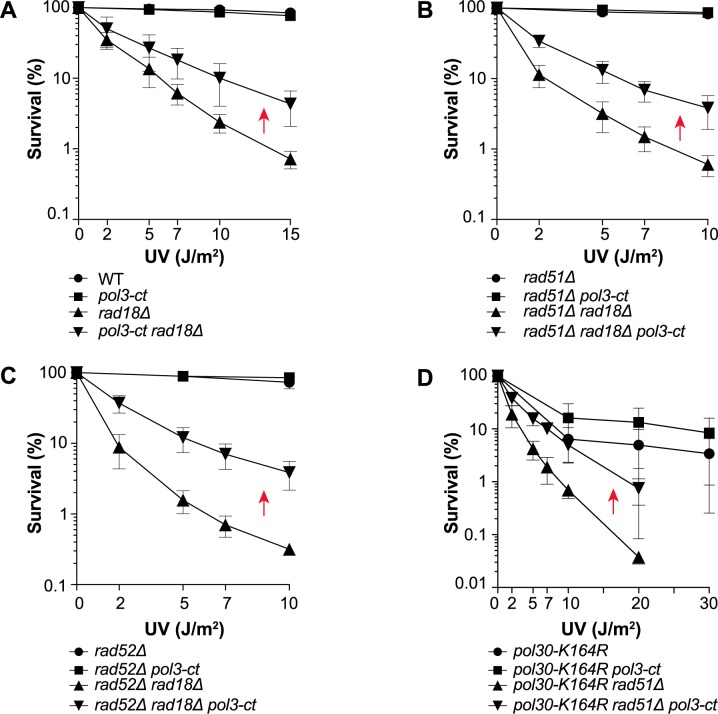
*pol3-ct* increases UV resistance of yeast cells deficient for PCNA ubiquitination independently of HR. Survival curves of haploid cells deficient for PCNA ubiquitination after exposure to UV light. Red arrows highlight increased UV resistance in the presence of *pol3-ct*. (A) Suppression of *rad18Δ* cells UV hypersensitivity by the *pol3-ct* allele. (B and C) The increased UV resistance of *rad18Δ pol3-ct* cells is HR-independent. (D) *pol3-ct* suppresses UV sensitivity of HR-deficient *pol30-K164R* cells.

In *rad18Δ* yeast mutant cells, UV-induced PCNA ubiquitination is abolished and the damage avoidance pathways are inhibited. Indeed, *rad18Δ* cells displayed UV hypersensitivity compared with WT cells (Fig 1A). Conversely, UV resistance was significantly increased in *rad18Δ* cells that carried also the *pol3-ct* allele of *POL3* (Fig 1A) in which the substitution of a Leu codon with a stop codon resulted in the loss of the four last C-terminal amino acids (LSKW) of Pol3 [58]. This mutation destabilizes the interaction between Pol3 C-terminal domain and Pol31 [59]. This result suggested that WT Pol δ could contribute to the UV sensitivity associated with *RAD18* deletion. To test this hypothesis, we performed a detailed genetic analysis.

### *pol3-ct* increases UV resistance in PCNA ubiquitination-deficient cells independently of homologous recombination

It has been already reported that *rad18Δ*-associated UV sensitivity is suppressed in the absence of the Srs2 helicase [60] or of the Siz1 SUMO ligase [61]. This suppression requires the homologous recombination (HR) genes *RAD51* and *RAD52*. However, in *rad18Δ* cells in which *RAD51* or *RAD52* was also deleted, UV resistance was increased in the presence of *pol3-ct* (Fig 1B and 1C). Therefore, we concluded that *rad18Δ* suppression by *pol3-ct* is mechanistically not related to HR. Moreover, *pol3-ct* did not suppress UV sensitivity associated with *rad51Δ* at high UV doses (S1A Fig). These findings suggested that *pol3-ct* effect could be specific to the DNA damage tolerance (DDT) pathways controlled by PCNA ubiquitination.

To determine whether *pol3-ct* suppressor effect was related to PCNA ubiquitination, we tested also the impact of *pol3-ct* in the *pol30-K164R* mutant. In this mutant, PCNA K164 is mutated, thereby preventing its ubiquitination by Rad6/Rad18 and sumoylation by Siz1. The *pol30-K164R* mutant was less sensitive to UV than the *rad18Δ* single mutant (Fig 1D). This phenotype can be explained by the lack of PCNA sumoylation in the *pol30-K164R* mutant that could lead to inefficient Srs2 recruitment [61,62] and consequently, to frequent channeling of UV-induced DNA lesions towards HR. Moreover, *pol30-K164R* UV sensitivity was not affected by *pol3-ct* (Fig 1D). Yet, in the absence of HR, *pol3-ct* had a clear suppressor effect (Fig 1D), thereby showing that *pol3-ct* restores UV resistance to both *pol30-K164R* and *rad18Δ* cells. Overall, our observations support the hypothesis that *pol3-ct* relieves partially the requirement of PCNA ubiquitination for UV resistance.

### *pol3-ct* increases UV resistance of mutants on the *RAD5-* and *MMS2-*dependent branch of the *RAD18* pathway

**Fig 2:**
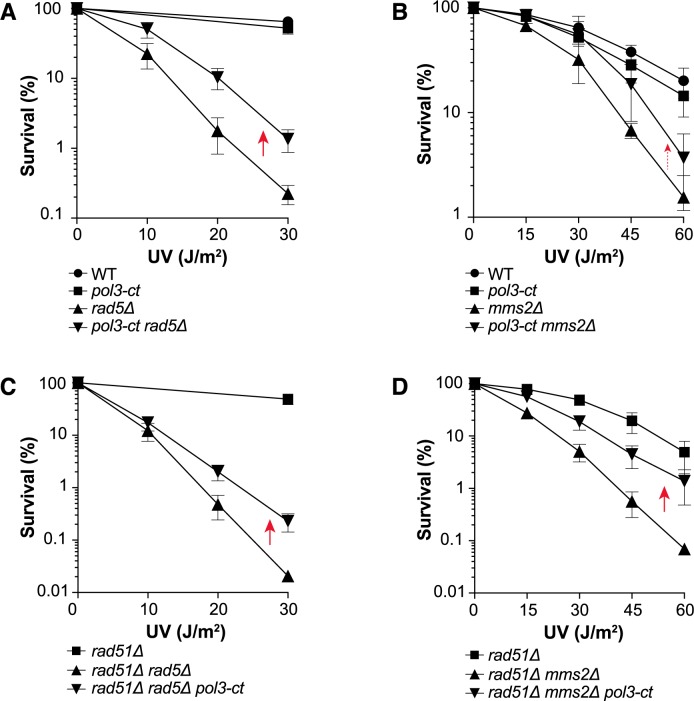
*pol3-ct* increases UV resistance in the absence of PCNA polyubiquitination. Survival curves of haploid cells deficient for PCNA poly-ubiquitination after exposure to UV light. Red arrows highlight increased UV resistance in the presence of *pol3-ct*. (A and C) Suppression of UV hypersensitivity in *rad5Δ* cells by the *pol3-ct* allele in the presence or absence of Rad51. (B and D) Suppression of *mms2Δ*-associated UV sensitivity by the *pol3-ct* allele in the presence or absence of Rad51. The weaker suppression of *mms2Δ* UV sensitivity by the *pol3-ct* allele is shown with a smaller and dotted red arrow.

Several DDT pathways are regulated by Rad18 (*i*.*e*., TLS activation and template switching). We noticed that *pol3-ct* suppressed the *rad18Δ* phenotype only partially and that *rad18Δ pol3-ct* cells remained sensitive to UV compared with WT cells (Fig 1A). This suggested that *pol3-ct* suppressor effect could involve mainly one of the Rad18-dependent pathways. To test this hypothesis, we first evaluated *pol3-ct* effect within the template switching pathway that is triggered by PCNA poly-ubiquitination catalyzed by Rad5 and Mms2-Ubc13. A *rad5Δ* mutant and, to a lesser extent, an *mms2Δ* mutant (both defective in PCNA poly-ubiquitination) regained some UV resistance in the presence of *pol3-ct* (Fig 2A and 2B). Moreover, both *rad5Δ* and *mms2Δ* strains showed a negative interaction with *rad51Δ* upon UV radiation, and the UV sensitivity of the *rad5Δ rad51Δ* and *mms2Δ rad51Δ* double mutants was similarly reduced by *pol3-ct* (Fig 2C and 2D). Thus, some of the UV-induced DNA lesions handled *via* template switching are bypassed in the *rad5Δ* and *mms2Δ* mutants thanks to *pol3-ct*.

**Fig 3:**
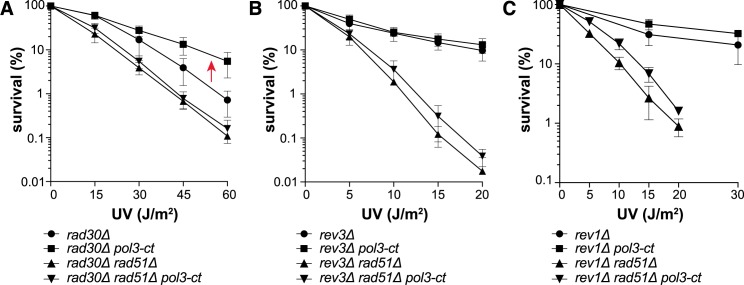
*pol3-ct* effects in cells lacking TLS polymerases. Survival curves of TLS-deficient haploid cells after exposure to UV light. (A) *pol3-ct* increases the UV resistance of *rad30Δ* cells (red arrow) only in the presence of Rad51. (B) *pol3-ct* does not increase UV resistance of *rev3Δ* cells in the presence of Rad51. A minor *pol3-ct* effect is observed in the absence of Rad51. (C) *pol3-ct* does not restore UV resistance of *rev1Δ* cells in the presence Rad51. A minor *pol3-ct* effect is observed in the absence of Rad51.

To bypass UV-induced lesions during DNA replication, Rad18 also controls the Pol η-dependent error-free and the Pol ζ- and Rev1-dependent error-prone TLS pathways. Concerning Pol η (encoded by *RAD30*), *pol3-ct* partially suppressed the UV sensitivity associated with the *rad30Δ* allele (Fig 3A). However, this effect was abolished in the absence of Rad51, suggesting a complex relationship between Pol δ-ct and Pol η (S7B Fig). *pol3-ct* had little effect on UV resistance in the Pol ζ and Rev1 mutants, even at higher UV doses (Fig 3B and 3C; S1B and S1C Fig). We noticed that in the *rev3Δ rad51Δ* and *rev1Δ rad51Δ* double mutants, *pol3-ct* had a minor suppressive effect, suggesting a modest Pol η recruitment (Fig 3B and 3C; S7C Fig). These observations imply that Pol ζ and Rev1 are still required for UV resistance in the *pol3-ct* mutant. As *pol3-ct* did not display a pervasive suppressor effect in TLS polymerase-defective mutants, we conclude that Pol δ-ct cannot take the place of TLS polymerases. Conversely, the *pol3-ct* suppressor effect clearly acts on the *RAD18*-dependent poly-ubiquitination sub-pathway. To explain these results, we hypothesized that Pol δ-ct reduces the PCNA ubiquitination requirement for TLS polymerase recruitment. In this model, suppression of the *rad18Δ* phenotype by *pol3-ct* should require the TLS polymerases.

### *rad18Δ* suppression by *pol3-ct* upon UV radiation depends on Pol ζ and Rev1

**Fig 4:**
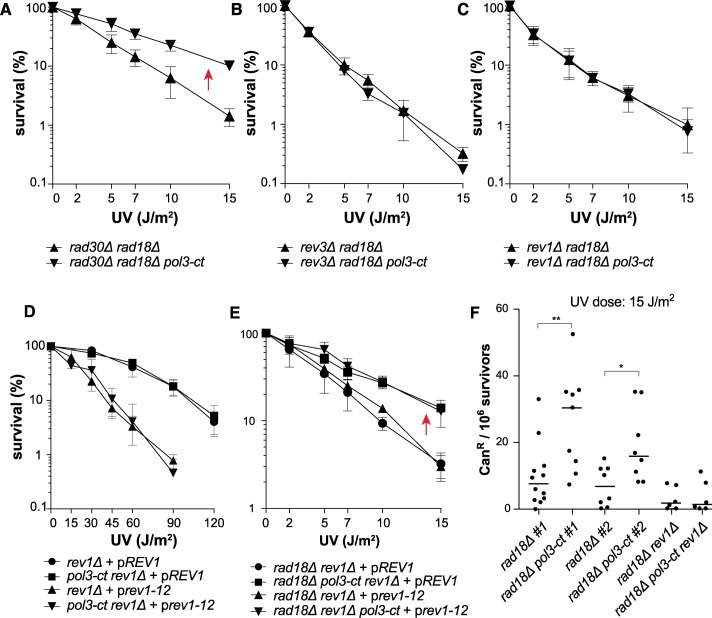
Suppression of *rad18Δ* UV phenotypes by *pol3-ct* requires *REV3* and *REV1*. Survival curves of UV-irradiated haploid cells with defective PCNA ubiquitination and TLS. (A) Suppression of *rad18Δ* UV sensitivity by *pol3-ct* (red arrow) does not require *RAD30*. (B) Suppression of *rad18Δ* UV sensitivity by *pol3-ct* requires *REV3*. (C) Suppression of *rad18Δ* UV sensitivity by *pol3-ct* requires *REV1*. (D) *pol3-ct* does not suppress the effect of the *rev1-12* allele. *rev1Δ* cells are complemented with a plasmid containing either the WT *REV1* gene (p*REV1*) or the *rev1-12* allele (p*rev1-12*). (E) Suppression of *rad18Δ* UV sensitivity by *pol3-ct* (red arrow) does not require a functional UBM domain in Rev1. *rad18Δ rev1Δ* cells are complemented with a plasmid containing either the WT *REV1* gene (p*REV1*) or the *rev1-12* allele (p*rev1-12*). (F) Increase of UV-induced mutagenesis in *rad18Δ* cells carrying *pol3-ct*. Each dot represents the can^R^ frequency measured in one experiment. The median value for each strain is represented by the horizontal bar. For this experiment, the *rad18Δ* and *rad18Δ pol3-ct* strains were isolated from two independent progenies (#1 and #2), and in both cases the can^R^ frequency between *rad18Δ* and *rad18Δ pol3-ct* was significantly different (Mann-Whitney test): **, *P* = 0.0043 (#1) and *, *P* = 0.0353 (#2).

The suppression of *rad18Δ* UV sensitivity by *pol3-ct* was still observed in the *rad30Δ* mutant (Fig 4A), which suggests a minor role for Pol η in the bypass of UV-induced DNA lesions in the *pol3-ct rad18Δ* double mutant. On the contrary, the *rev3Δ rad18Δ pol3-ct* and *rev1Δ rad18Δ pol3-ct* strains were UV hypersensitive, like the *rev3Δ rad18Δ* and *rev1Δ rad18Δ* double mutants (Fig 4B and 4C). Thus, suppression of *rad18Δ* UV sensitivity by *pol3-ct* occurs only when Pol ζ and Rev1 are present. Interestingly, we observed that suppression of *rad5Δ-*associated UV sensitivity by *pol3-ct* was dependent on both Pol ζ and Pol η (S2 Fig), showing that Pol η can play a role in *pol3-ct* strains when PCNA is ubiquitinated.

Rev1 carries a conserved UBM motif in its C-terminus. As *pol3-ct* relieves partially the requirement for PCNA ubiquitination, the Rev1 UBM should be dispensable in the *pol3-ct* mutant. Point mutations within this motif (L821A, P822A, I825A) abolish its functional interaction with ubiquitinated PCNA *in vitro* and strongly lower cell resistance to UV radiation *in vivo* [29]. In the *pol3-ct* background, UV sensitivity was not reduced in the *rev1-12* mutant that carries the L821A, P822A, I825A mutations, (Fig 4D). However, the *rad18Δ* phenotype was suppressed by *pol3-ct* in the *rev1-12* mutant (Fig 4E). Thus, we conclude that in the *pol3-ct* mutant, Pol ζ and Rev1 bypass UV-induced DNA lesions in the absence of PCNA ubiquitination independently of Rev1 UBM.

UV-induced mutagenesis mostly depends on Pol ζ and Rev1 and on PCNA ubiquitination [8,63–65]. In the *pol3-ct* mutant, the role of PCNA ubiquitination in UV-induced mutagenesis could be less important. To test this hypothesis, we monitored UV-induced mutagenesis with the *CAN1* forward mutation assay after exposure to a UV dose of 15 J/m^2^. At this dose, only about 1% of *rad18Δ* cells survived and *pol3-ct* strongly suppressed *rad18Δ*-associated UV sensitivity (Fig 1A). Interestingly, we observed that the frequency of UV-induced canavanine resistant (Can^R^) cells was higher in the *rad18Δ pol3-ct* double mutant compared with *rad18Δ* cells (Fig 4F). This result was obtained with strains coming from two different progenies and is no longer observed in absence of Rev1 (Fig 4F). Thus, these observations further support the hypothesis that upon UV irradiation, Pol ζ and Rev1 are recruited independently of PCNA ubiquitination in the *pol3-ct* mutant.

### Pol3 and Pol31 mutations that affect the Pol3-CTD/Pol31 complex like Pol3-ct

**Fig 5:**
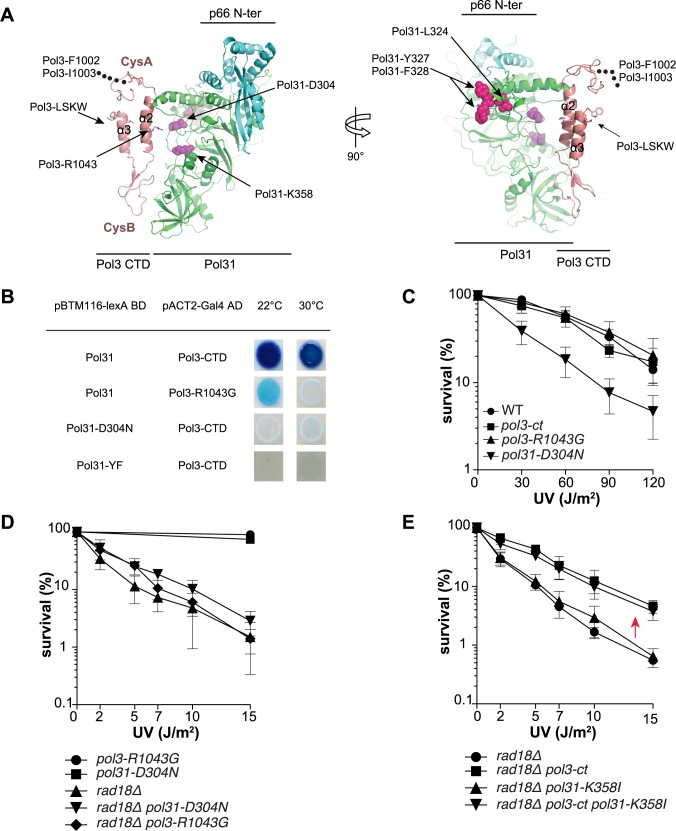
Functional analysis of the interaction between Pol3-CTD and Pol31 in cell survival after UV irradiation. (A) Structural model of the Pol3-CTD/Pol31/P66 complex. Pol3 CTD contains two conserved cysteine-rich metal-binding motifs (CysA and CysB) that are separated by two α-helices. The Arg1043 residue of Pol3 lies within helix α2 between the Pol3 LSKW residues and Pol31. In addition, Pol31 Asp304 is very close to Pol31 interacting surface opposite to Pol3 Arg1043. The position of the LSKW motif at the C-terminal end of Pol 3 CTD is indicated by Pol3-LSKW. The conserved L1094 and W1097 residues of this motif are indicated by sticks. This motif is deleted in the *pol3-ct* mutant. The Pol3 residue R1043 within helix α2 is also indicated by sticks. The Pol3 residues F1002 and I1003 are located five amino-acids upstream of C1009 (the first amino-acid included in the structural model). These five amino-acids are indicated by black dots to show the proximity of Pol3-F1002 and -I1003 with Pol3-C1009. Pol31-D304 and -K358 residues are highlighted as magenta spheres. Pol31-L324, -Y327 and -F328 residues are shown as red spheres. Note that Pol31-L324 is buried in our model and not available for intermolecular interactions. (B) Yeast two-hybrid assays were performed using pBTM116 plasmids carrying WT and mutated Pol31 fused to the lexA binding domain (BD) and pACT2 plasmids carrying WT or mutated Pol3 C-terminal amino acids 1032–1097 fused to the Gal4 activating domain (AD). Each spots illustrates an individual co-transformant obtained following transformation of CTY10-5d. β-galactosidase activity was tested using an overlay plate assay at 22°C and 30°C. (C) Survival curves obtained after UV-irradiation of Pol δ mutant strains in which the interaction between Pol3 CTD and Pol31 is impaired. (D and E) Survival curves obtained after UV-irradiation of PCNA ubiquitination-deficient Pol δ mutant strains. (D) The *pol3-R1043G* and *pol31-D304N* alleles do not suppress *rad18Δ*-associated UV sensitivity. (E) The *pol31K358I* allele does not affect the suppression of *rad18Δ*-associated UV sensitivity by *pol3-ct* (red arrow).

Pol3-ct interacts weakly with Pol31 [59]. To determine whether this interaction plays a role in PCNA ubiquitination requirement, we wanted to identify mutations that affect this interaction and then test them genetically in the *rad18Δ* mutant. Based on our previous three-dimensional model of the Pol3-CTD and Pol31 complex from the published structures of human p50 and of Pol α [59,66,67] (Fig 5A), we focused our analysis on Arg1043 of Pol3 and Asp304 of Pol31, which is opposite to Pol3 Arg1043 (Fig 5A). Two-hybrid analyses [59] indicated that co-transformants carrying pBTM116-Pol31 and pACT2-Pol3-R1043G displayed temperature-sensitive β-galactosidase activity after incubation for two days. Compared with the activity of pBTM116-Pol31 and pACT2-Pol3-CTD co-transformants, their activity was low at 22°C and even lower at 30°C, showing that Pol3-R1043G gives similar results than Pol3-ct (Fig 5B; [59]). Co-transformants carrying pBTM116-Pol31-D304N and pACT2-Pol3-CTD did not show any measurable β-galactosidase activity at either temperature (Fig 5B).

*pol3-R1043G* did not confer sensitivity to the genotoxic agent hydroxyurea (HU) differently from *pol3-ct* (S3A Fig). Yet, we observed synthetic lethality between *pol3-R1043G* and *pol32Δ*, a phenotype shared with *pol3-ct* (Material & Methods; S4 Fig). The *pol31-D304N* allele (originally named *hys2-2*; [68]) conferred temperature and HU sensitivities (S3A Fig) and was synthetic lethal with *pol32Δ* (S4 Fig). In summary, the Pol3-R1043G mutation affected the structure of the Pol δ holoenzyme although to a lesser extent than the Pol3-ct and the Pol31-D304N mutations.

### Partial loss of interaction between Pol3 CTD and Pol31 does not promote Rad18-independent TLS

To determine the role of the interaction between Pol3 CTD and Pol31 in bypassing PCNA ubiquitination, we first evaluated UV sensitivity in the *pol3-ct*, *pol3-R1043G* and *pol31-D304N* strains. The *pol3-ct* and *pol3-R1043G* mutants were not UV sensitive and showed UV-induced mutagenesis frequencies similar to those of WT cells (Fig 5C; S5 Fig). Conversely, the *pol31-D304N* allele increased UV sensitivity (Fig 5C). As Pol31 is a member of the Pol ζ complex, we hypothesized that this phenotype could be caused by a partial impairment of the interaction between Pol31 and Rev3 CTD. Interestingly, UV-induced mutagenesis in the *pol31-D304N* mutant was significantly decreased, but not abolished (S5 Fig).

Based on the UV phenotypes of Pol δ mutants, we hypothesized that *rad18Δ* UV hypersensitivity could be suppressed by *pol3-R1043G*, but not by *pol31-D304N* due to its possible defect in the Pol ζ-dependent pathway. However, neither *pol3-R1043G* nor *pol31-D304N* suppressed the *rad18Δ* phenotype (Fig 5D). Thus, in disagreement with our initial prediction, suppression of *rad18Δ* UV sensitivity by *pol3-ct* might not be related to a defective interaction between Pol3 CTD and Pol31. To test genetically this hypothesis, we used the *pol31-K358I* allele that suppresses *pol3-ct* effect [59]. K358 is accessible for interaction with Pol3 CTD residues (Fig 5A). The *pol31-K358I* allele did not modify the suppression of *rad18Δ* UV hypersensitivity by *pol3-ct*, reinforcing the conclusion that *pol3-ct* effect on *rad18Δ* is not related to the interaction between Pol3 CTD and Pol31 (Fig 5E).

### Among the PIP motifs in the three subunits of Pol δ, only Pol32 PIP motif interacts with PCNA

As an alternative hypothesis, TLS polymerase recruitment could be facilitated by Pol δ mutations that weaken its interaction with PCNA. Our strategy was again to find these mutations and to test them subsequently in *rad18Δ* strains. However, this search was hampered by the lack of a ternary structure of PCNA complexed with Pol δ on DNA. Hence, we focused on Pol δ sites that were described as PIP motifs [52,53]. Pol32 PIP motif (**Q**xx**L**xx**FF)** contains the PIP consensus sequence, is highly conserved in eukaryotes [52] (S6C Fig) and has been implicated in PCNA interaction by two-hybrid screens [52]. The Pol31 motif **L**xx**YF** lacks the conserved Gln residue, is not conserved and is located in the Pol31 PDE domain [53,67] (S6B Fig). In our Pol31 model, the aromatic dyad is exposed and the preceding L324 could be buried (Fig 5A). The Pol3 motif **Q**xxx**L**xx**F**I differs from the consensus PIP motif by the presence of an extra residue between the conserved Gln and Leu residues and by the lack of the second aromatic residue at the end of the motif. Moreover, this motif is located just upstream of the highly conserved CysA cysteine module (Fig 5A), and only the Leu and Phe residues are conserved in eukaryotes (S6A Fig).

**Fig 6:**
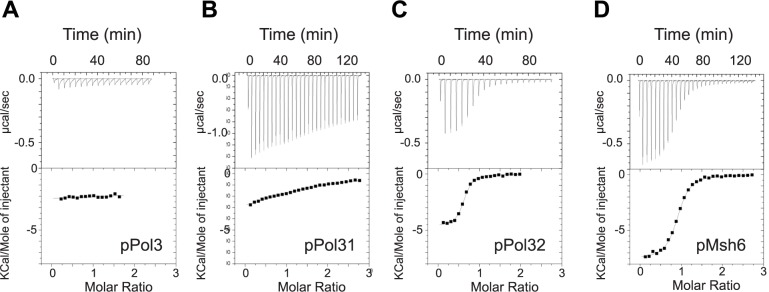
Physical interactions between PCNA and the proposed PIP-boxes of *S*. *cerevisiae* Pol32, Pol31 and Pol3. The thermograms (upper panels) and binding isotherms (lower panels) of the calorimetric titrations of PCNA by the assayed peptides at 303 K are presented. The corresponding thermodynamic parameters are reported in Table 1. (A, B, C, D) Interaction between PCNA and the peptides containing the (putative and canonical) Pol3, Pol31, Pol32 and Msh6 PIP motifs, respectively. The thermodynamic parameters ΔH, N, and Ka were obtained by non-linear least-squares fitting (represented in line mode) of the experimental data with a single site model.

To test the functionality of these three motifs, we characterized by microcalorimetry the affinity and stoichiometry of the interaction between purified PCNA and synthetic peptides that contain the putative PIP-boxes found in Pol δ. The Pol3 and Pol31 peptides were of equal length (21 amino acids), whereas the Pol32 PIP motif, which is located in the extreme C-terminus of Pol32, was three amino acids shorter. In parallel, we tested the canonical PIP motif of Msh6 that mediates the interaction of MutSα with PCNA [69]. The binding reactions between Msh6 and Pol32 peptides with PCNA gave large exothermic signals and the interactions could be fitted with a one-site binding model after integration. The dissociation constant (Kd) of the Msh6 and Pol32 PIP motifs were 0.31 μM and 0.46 μM at 30°C, respectively (Table 1, Fig 6C and 6D). Both interactions presented favorable enthalpy and entropy, as previously observed for other canonical PIP motifs [14,70,71]. In the same experimental condition, the non-canonical Pol3 and Pol31 PIP motifs showed no interaction or very weak interaction, respectively (Fig 6A and 6B).

**Table 1:** Thermodynamic parameters of the interactions between PCNA and the proposed Pol32, Pol31 and Pol3 PIP motifs measured by calorimetry.

Peptide	Sequence	Length (aa)	Kd (μM)	ΔH° (kcal/mol)	-TΔS° (kcal/mol)	ΔG° (kcal/mol)
pPol3 992–1012	TGS**Q**KGG**L**MS**FI**KKVEACKSC	21	NI	NI	NI	NI
pPol31 317–337	KSLFDKS**L**ES**YF**NGSNKEILN	21	>50	nd	nd	nd
pPol32 333–350	KRLKK**Q**GT**L**ES**FF**KRKAK^a^	18	0.46 ± 0.15	-4.45 ± 0.8	-4.65 ± 1.0	-9.05 ± 0.2
pMsh6 22–43	QKKMK**Q**SS**L**LS**FF**SKQVPSGTP	26	0.31 ± 0.1	-6.40 ± 1.0	-2.39 ± 1.06	-8.8 ± 0.2

All ITC experiments were done at 30°C. Data are shown as the mean value ± standard deviation of at least two measurements. The amino acids of the proposed PIP motifs are in bold underlined characters. The numbers under the peptides’ names indicate to the amino acid position in the sequence of the corresponding protein. The Kd for Pol31 corresponds to a lower value estimated from the weak signal observed with this peptide. The thermograms and isotherms are reported in Fig 6. NI: no interaction; nd: not determined.

^a^K is the last residue of Pol32.

In summary, while the Pol32 PIP motif has the characteristics of a functional PIP motif, the proposed Pol3 and Pol31 non-canonical motifs may have a role in Pol δ function or in Pol δ interaction with PCNA, but possibly not as *bona fide* PIP domains.

### The viability of the triple *pol3-FI pol31-YF pol32-pip* mutant implies a functional PCNA-Pol δ interaction in this mutant

Although they do not interact with PCNA, the Pol3 and Pol31 non-canonical PIP motifs contribute to PCNA-stimulated DNA synthesis *in vitro* [53]. To study their role *in vivo*, we mutated the Pol3 **Q**xxx**L**xx**F**I and Pol31 **L**xx**YF** motifs and generated strains that carry either the *pol3-FI1002-1003AA* allele (thereafter, named *pol3-FI*) or the *pol31-YF327-328AA* allele (thereafter, named *pol31-YF*). We also produced the *pol32-FF344-345LL* allele (thereafter, named *pol32-pip*) by directed mutagenesis of the Pol32 PIP motif. Mutants carrying these alleles did show neither slow growth, nor temperature or HU sensitivity (S3 Fig). In addition, all double mutants were viable and thermo-resistant. Only the *pol3-FI pol32-pip* double mutant was sensitive to HU (S3B Fig). Importantly, the *pol3-FI pol31-YF pol32-pip* triple mutant was viable, strongly suggesting that in this mutant, PCNA is still a processivity factor for Pol δ (S3B Fig). Moreover, the triple mutant was more sensitive to HU than the *pol3-FI pol32-pip* double mutant. This suggests that *pol31-YF* has some effect on Pol δ structure stability. Accordingly, *pol31-YF* showed synthetic lethality with *pol32Δ* (S4 Fig) and an additive effect with *pol3-ct* upon HU treatment (S3A Fig). Finally, the Pol31 **L**xx**Y**F motif is close to the Pol31-D304 residue involved in the interaction with Pol3 CTD (Fig 5A). By two-hybrid assay, we found that the Pol31 YF residues were as important for this interaction as the D304 residue (Fig 5B). This also suggests that these residues contribute to the overall stability of the Pol δ holoenzyme.

### *rad18Δ*-associated UV hypersensitivity is partially suppressed by mutational inactivation of the Pol3 F1002-I1003 residues upstream of the Pol3 CysA module

**Fig 7:**
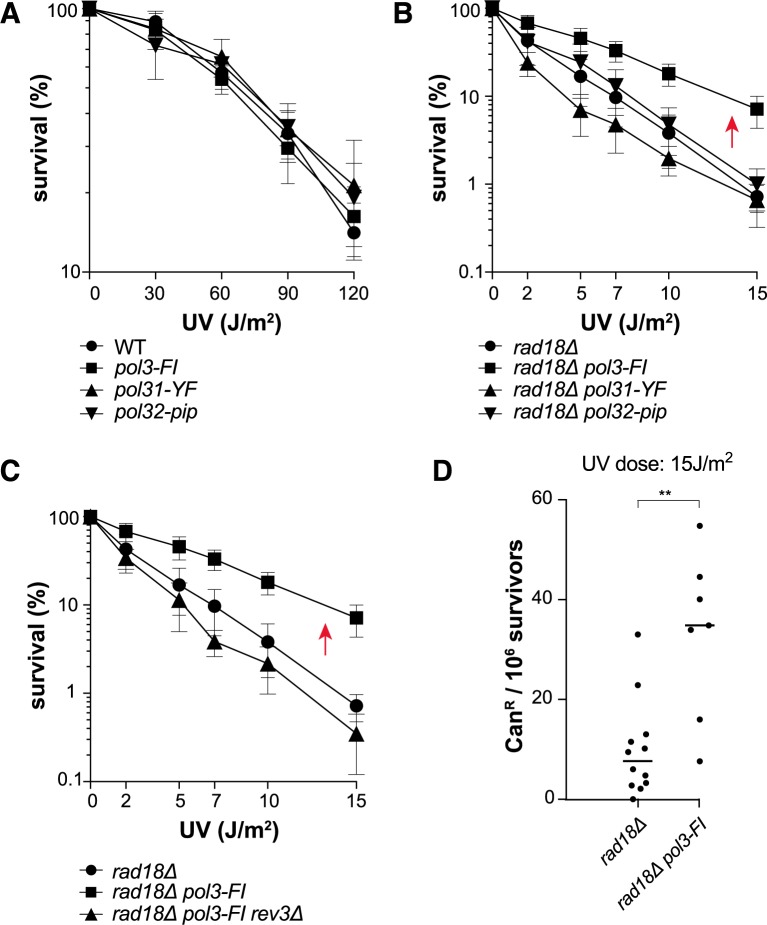
Suppression of *rad18Δ* UV phenotypes by *pol3-FI*. (A) Survival curves of UV-irradiated mutant strains carrying the *pol3-FI*, *pol31-YF* or *pol32-pip* allele. (B) *pol3-FI* suppresses *rad18Δ*-associated UV sensitivity (red arrow), while *pol31-YF* and *pol32-pip* do not. (C) Suppression of *rad18Δ*-associated UV sensitivity by *pol3-FI* (red arrow) depends on *REV3*. (D) Increase of UV-induced mutagenesis in *rad18Δ* cells carrying *pol3-FI*. Each dot represents the can^R^ frequency measured in one experiment. The median for each strain is represented by a horizontal bar: **, *P* = 0.0026 (Mann-Whitney test).

UV resistance (Fig 7A) and UV-induced mutagenesis (S5 Fig) were comparable in the three *pol3-FI*, *pol31-YF* and *pol32-pip* single Pol δ mutants and in WT cells. Thus, these mutants carry a functional Pol ζ. Therefore, we could test the role of the Pol δ mutated motifs in the absence of PCNA ubiquitination. UV sensitivity was comparable in the *pol32-pip rad18Δ* double mutant and in the *rad18Δ* single mutant (Fig 7B). Thus, the Pol32-pip mutation did not affect the competition for PCNA access between Pol δ and Pol ζ. We obtained similar results with the *pol31-YF rad18Δ* mutant (Fig 7B). Conversely, *pol3-FI* suppressed *rad18Δ-*associated UV hypersensitivity and this effect was *REV3* dependent (Fig 7C). In agreement with these observations, UV-induced mutagenesis was significantly increased in *rad18Δ pol3-FI* cells compared with *rad18Δ* cells (Fig 7D). Thus, only *pol3-FI* recapitulates the phenotypes of the *pol3-ct* allele in the *rad18Δ* background. The Pol3 FI residues (mutated in the *pol3-FI* strain) and Pol3 LSKW residues (lost in the *pol3-ct* strain) are close to the CysA module of Pol3-CTD in our structural model (Fig 5A). Therefore, the phenotypes shared by *pol3-ct* and *pol3-FI* could be due to destabilization of the CysA module that has been proposed to be essential for the Pol δ-PCNA interaction ([50], Discussion).

## Discussion

In the present study, we showed that Pol ζ and Rev1 can be activated *in vivo* independently of PCNA ubiquitination in the *pol3-ct* and *pol3-FI* mutants. From this genetic observation, we infer that the primary role of PCNA ubiquitination is to promote the recruitment of TLS polymerases that would otherwise be outcompeted by Pol δ for access to the front side of PCNA and to the 3’ primer ends, even in the presence of UV-induced DNA lesions leading to fork stalling. In the next paragraphs, we will emphasize that the suppression of *rad18Δ* cells UV sensitivity by these Pol δ mutants is novel and robust. Then, we will discuss the possible mechanism by which this suppression occurs. We will argue that it is not mediated through the easier replacement of Pol3 by Rev3/Rev7 on a putative Pol31/Pol32 platform, but through the partial loss of interaction between Pol3 and PCNA. Finally, we will present a working model that takes into account the finding that *rad18Δ* suppression by *pol3-ct* and *pol3-FI* occurs mostly through Pol ζ and Rev1 and not through Pol η.

### *pol3-ct* and *pol3-FI* are novel suppressors of *rad18Δ*

The *pol3-ct* and *pol3-FI* alleles do not confer UV sensitivity to WT cells or to cells with defects in one or several UV-induced DNA lesion tolerance pathways ([59], Figs 5C and 7A, S1 Fig). Moreover, these alleles are not associated with a defect in UV-induced mutagenesis (S5 Fig). These key features allowed us to detect the partial rescue of the *rad18Δ* mutant by *pol3-ct* and *pol3-FI* following UV radiation. Although *rad18Δ* still confers high UV sensitivity in the presence of the *POL3* alleles (Figs 1A and 7B), we considered the phenotypic suppression robust for the following reasons. At higher UV doses, *rad18Δ* cell survival is increased at least ten-fold in the presence of the *pol3-ct* and *pol3-FI* alleles. *pol3-ct* suppresses the *rad18Δ* phenotype in the W303 and the FF genetic backgrounds and in several genetic contexts (*pol30-K164R*, *rad5Δ* and *mms2Δ*). Finally, this suppression seems to occur only through the error-prone repair pathway, which explains why *rad18Δ* cells still retain high UV sensitivity. In addition, *pol3-ct* suppression of *rad18Δ* UV sensitivity is novel. It does not require functional HR, differently from the *srs2Δ*- and *siz1Δ-*dependent suppressions [60,61]. Moreover, studies based on the inactivation of the Pol δ proofreading domain in yeast and DT40 cells indicated that Pol δ contributes to TLS *in vivo* [72,73]. This propensity of the exonuclease-dead Pol δ to perform TLS in yeast is independent of Pol ζ and remarkably suppresses also *rad18Δ-*associated hypersensitivity to DNA damaging agents [72]. On the other hand, *rad18Δ* suppression by *Pol3-ct* and *pol3-FI* depends on Pol ζ and Rev1. Our results allow us to revisit the role of PCNA ubiquitination and of polymerase switch upon UV radiation.

### Pol ζ recruitment independently of PCNA ubiquitination might not occur through a switch between Pol3 and Rev3/Rev7 on a Pol31/Pol32 platform

How to explain that Pol δ-ct and Pol δ-FI allow the recruitment of Rev1 and Pol ζ independently of PCNA ubiquitination? The finding that Pol ζ may function as a four-subunit enzyme (Pol ζ4) has led to the suggestion that the polymerase switch involves dissociation of the Pol δ catalytic subunit (Pol3 in yeast) from its structural subunits (Pol31 and Pol32 in yeast), which will become part of Pol ζ [54–56]. In addition, DNA damage and PCNA ubiquitination trigger Def1-dependent degradation of the Pol3 catalytic subunit of yeast Pol δ [57]. Def1-dependent Pol3 degradation could be the initial event leading to polymerase switching on the Pol31/Pol32 platform [57]. The genetic suppression of *rad18Δ* by *pol3-ct* and *pol3-FI* described here depends on Pol ζ. In addition, *pol3-ct* partially impairs the interaction between Pol3 and Pol31 [59]. These observations fit well with a model in which in our *pol3* mutants, the switch between Pol3 and Rev3/Rev7 on the Pol31/Pol32 platform occurs spontaneously without the need of PCNA ubiquitination and the subsequent Pol3 poly-ubiquitination by Def1.

Yet, and rather unexpectedly, the results of our genetic analyses imply that the impaired interaction between Pol3 and Pol31 is not involved in the suppression of *rad18Δ*. The Pol3-R1043G mutant protein displays the same defect as the pol3-ct mutant in the two-hybrid interaction with Pol31, and both *pol3-R1043G* and *pol3-ct* are synthetic lethal with *pol32Δ*. However, *pol3-R1043G* does not suppress *rad18Δ* (Fig 5D). Similarly, the simultaneous mutational inactivation of Pol31 Y327 and F328 impairs the interaction between Pol31 and Pol3-CTD (Fig 5B) and leads to synthetic lethality with *pol32Δ* (S4 Fig). However, the *pol31-YF* mutant allele does not suppress *rad18Δ* (Fig 7B). The Pol31-K358I mutation restores stable Pol3-Pol31 interactions in the *pol3-ct* mutant and *pol31-K358I* suppresses *pol3-ct*-associated HU sensitivity [59]. On the contrary, *pol31-K358I* does not affect *rad18Δ* suppression by *pol3-ct* (Fig 5E). Finally, the *pol3-FI* allele suppresses *rad18Δ*, although the Pol3 F1002 and I1003 residues are upstream of the C-terminal Pol3 domain that is sufficient for interaction with Pol31 [74] (Fig 7C). Thus, the model implying a switch between Pol3 and Rev3/Rev7 on a Pol31/Pol32 platform does not fully account for the bypass of PCNA ubiquitination observed in the *pol3* mutants.

### A partial loss of interaction between Pol3 CTD and PCNA might allow the suppression of *rad18Δ*-associated UV hypersensitivity

These observations suggest that the effect of the Pol3-ct mutation on Pol δ structure does not impair only the interaction with Pol31. Therefore, we hypothesized that *pol3-ct* destabilizes the interaction between Pol δ and PCNA. Hence, we expected to find Pol δ mutations that affect this interaction and that would share the *pol3-ct* phenotypes. However, the lack of a ternary structure that includes Pol δ bound to PCNA at primer ends made our search uncertain. Moreover, Pol δ interacts with PCNA through multiple sites and a possible redundancy in binding interactions could allow Pol δ to adopt flexible configurations with PCNA [75,76]. Our understanding of PCNA-Pol δ interactions is even more challenged by our finding that among the three proposed PIP motifs of Pol δ, only the Pol32 motif is a *bona fide* PIP motif (Table 1, Fig 6A)[53]. The observation that the *pol3-FI pol31-YF pol32-pip* triple mutant is viable in our strain backgrounds (W303 and FF18733; S4 Fig and S1 Table) clearly indicates that Pol δ interacts with PCNA in this triple mutant.

Despite the uncertainties surrounding PCNA-Pol δ interaction, the Pol3 F1002 and I1003 residues are separated by only five amino-acids from Pol3 C1009, the first cysteine of the CysA zinc-binding segment of Pol3 CTD (Fig 5A). This CysA motif is likely to be crucial for the interaction with PCNA [50]. Thus, Pol3 F1002 and I1003, while not directly required for the interaction with PCNA, might contribute to the optimum structural conformation of Pol3 CysA. Destabilization of this CysA module could affect the interaction between Pol δ and PCNA and consequently facilitate the recruitment of TLS polymerases to PCNA. To substantiate this hypothesis, we wanted to mutate the CysA cysteines (C1009, C1012 or C1024), but failed. This suggests that these cysteines are essential and that the CysA module is crucial for PCNA-Pol δ complex stability [50,77]. The last Pol3 C-terminal W1097 residue is close to the CysA module and therefore, could play a role in its stabilization as well (Fig 5A). Thus, the loss of the last four LSKW residues in the *pol3-ct* mutant might affect the interaction with Pol31 and also with PCNA. Our genetic observations support a role for Pol3 F1002 and I1003 and for Pol3 W1097 in promoting stable interactions between Pol3 and PCNA. Only *pol3-ct* and *pol3-FI* suppress the UV hypersensitivity associated with *rad18Δ* and remarkably, only these two *POL3* alleles display an additive negative effect with the *pol32-pip* allele upon HU treatment (S3 Fig). Similarly, when both Pol3 CysA and Pol32 PIP motifs are mutated, the PCNA-dependent replication activity of Pol δ is almost abolished, showing an additive negative effect between these sites *in vitro* [50]. Therefore, we propose that the impaired interaction between a Pol3 CTD mutant and PCNA is the origin of the suppression of *rad18Δ*-dependent UV sensitivity.

### Model for PCNA ubiquitination-dependent polymerase switch at UV-induced DNA lesions

**Fig 8:**
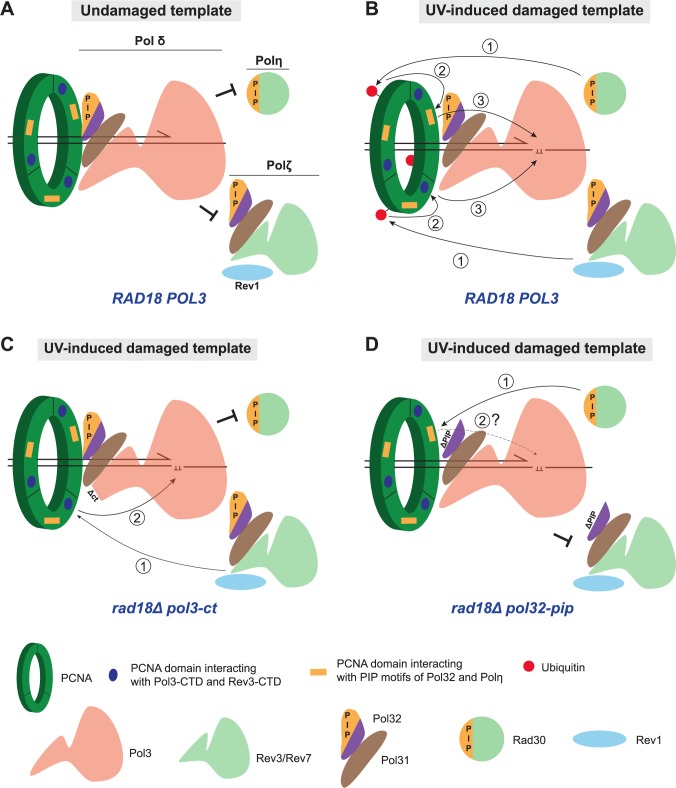
Polymerase switching in Pol δ structural mutants: A working model. (A) DNA synthesis on an undamaged DNA template is catalyzed by Pol δ. Thanks to PCNA trimeric structure, simultaneous binding of two or even three Pol δ molecules could be envisaged. This possibility is not depicted here for the sake of simplicity. Pol δ outcompetes TLS polymerases for the access to the 3’ DNA primer terminus by stronger affinity or mass action. Pol δ subunits interact with several PCNA domains. The PCNA domain interacting with PIP motifs is represented by an orange rectangle on PCNA, whereas the one interacting with the CysA module of Pol3-CTD and possibly with the CysA module of Rev3-CTD is represented by a blue filled circle (note that this PCNA domain has not been identified yet). (B) Pol δ stalls when the 3’ end of the newly synthesized strand encounters a UV-induced DNA lesion such as a pyrimidine dimer. Rad6/Rad18 activation triggers PCNA ubiquitination at K164 (red filled circle). TLS polymerases access to the 3’ end is envisaged in three steps: 1) the TLS polymerase-ubiquitin interaction brings these polymerases closer to their PCNA interacting domain and therefore increases their local concentration near the 3’ end; 2) thanks to this proximity, TLS polymerases can compete with Pol δ for binding to their specific PCNA interaction domain: PIP interacting domain (orange rectangle) for Pol η and a not-yet-identified PCNA domain (blue filled circle) for Pol ζ *via* its CysA module. Following its displacement, Pol δ disengages from binding to the primer terminus; 3) tight binding of TLS polymerases on the front face of PCNA allows the insertion of the 3’ extending end and the template UV-induced DNA lesion into the catalytic site of TLS polymerases for translesion synthesis. (C) Pol δ-ct (and Pol3-FI) leads locally to the destabilization of the Pol3-CTD-PCNA interaction. With the help of Rev1, this could facilitate Pol ζ competition for the common PCNA interacting domain. Thus, the initial binding step to ubiquitinated PCNA is bypassed and Pol ζ has access to the 3’ end in two steps only: 1) Pol ζ binds to its PCNA interacting domain (blue filled circle); and then 2) Translesion synthesis takes place. Conversely, Pol η does not have access to its PCNA interacting domain in *pol3-ct*. (D) When Pol δ carries the mutant Pol32-ΔPIP subunit, Pol η PIP domain should readily interact with PCNA PIP interacting domain (orange rectangle, step 1) and the requirement of PCNA ubiquitination should be bypassed. This hypothesis was proved to be false because the *pol32-pip* allele does not suppress UV sensitivity associated with *rad18Δ*. Thus, Pol η cannot take over DNA synthesis at UV-induced DNA lesions (step 2 indicated by a dashed arrowed line and a question mark). Pol η might require additional PCNA interactions to accommodate pyrimidine dimers on its catalytic site.

The observation that *pol3-ct* and *pol3-FI* suppress *rad18Δ*-associated UV hypersensitivity leads to the conclusion that WT Pol δ is in part responsible for this sensitivity. In addition, our data suggest that Pol δ competes with TLS polymerases for the access to primer/template junctions in the front side of PCNA (Fig 8A). Therefore, PCNA ubiquitination might provide a docking site on the back side of PCNA for recruiting TLS polymerases, thereby increasing their local accumulation near a stalled 3’ end (Fig 8B). Spontaneous or active Pol δ displacement from its interaction domains with PCNA at sites of DNA damage would be accompanied by the concomitant binding of a TLS polymerase to the front side of PCNA and its access to the primer end. In the absence of PCNA ubiquitination, preferential binding of Pol δ to the front side of PCNA due to stronger affinity or mass action would inhibit the polymerase switch at blocking DNA lesions.

Our model is based on the activation of Pol ζ and Rev1 independently of PCNA ubiquitination observed in the *pol3-ct* and *pol3-FI* mutants. A noticeable issue in our model is the absence of a similar activation of Pol η in *rad18Δ* cells. From this unexpected result, we hypothesized that the key factor of *rad18Δ* suppression might be the specific interaction between Pol η or Pol ζ with PCNA (Fig 8B). Given the structurally conserved organization of the catalytic Pol3 and Rev3 subunits [50], the CysA metal-binding motif in Rev3 CTD and the CysA motif of Pol3 might interact with the same PCNA region. Thus, upon destabilization of Pol3 CTD, PCNA ubiquitination could be bypassed only through the binding of Rev1 and Rev3 CTD to PCNA (Fig 8C). Differently from Pol ζ, Pol η relies on a PIP motif for its interaction with PCNA and does not carry a metal-binding motif in its CTD. If the PCNA domain for interaction with CysA modules were different from the domain that interacts with the PIP motifs, Pol η would not be able to compete with Pol δ even when Pol δ harbors a mutated CTD (Fig 8C). According to this hypothesis, *pol32-pip* should suppress *rad18Δ*-associated UV sensitivity in a Pol η-dependent manner (Fig 8D). However, this prediction is not supported by our results suggesting that Pol η requires additional PCNA-dependent engagement at the primer terminus to incorporate distorting DNA lesions, such as pyrimidine dimers, into its catalytic site. To test this hypothesis, it will be important to investigate the suppression of *rad18Δ*-associated phenotypes by new Pol δ structural mutants or at DNA lesions different from those induced by UV exposure.

### A regulatory role for Pol δ in DNA damage tolerance

Our results also bring some insights into Pol δ regulatory role at stalled replication forks with ubiquitinated PCNA (S7 Fig). The *pol3-ct* allele does not increase UV-induced mutagenesis and is not associated with UV sensitivity. Thus, the *pol3-ct* effect on the choice of DDT pathway might be too subtle to be detected in the presence of PCNA ubiquitination (S7A Fig). However, the *rad30Δ* strain carrying *pol3-ct* showed higher UV resistance, and this effect was abolished in the *rad30Δ rad51Δ pol3-ct* mutant (Fig 3A). This observation suggests that the Pol δ-ct defect might facilitate the channeling of UV-induced DNA lesions processed by Pol η towards the HR pathway (S7B Fig). We observed a minor *pol3-ct*-dependent suppression of UV sensitivity with the *rev3Δ* strains (S1B Fig) and with the *rev3Δ rad51Δ* and the *rev1Δ rad51Δ* strains (Fig 3B and 3C). These last observations suggest an easier Pol η recruitment in competition with Pol δ-ct (S7C Fig). This hypothesis is supported by the clear suppression of the *rad5Δ*- and *mms2Δ*-associated UV sensitivity by *pol3-ct* (Fig 2) that depends on Pol ζ but also on Pol η (S2 Fig; S7D Fig). Pol η would be more suitable to displace the mutant Pol δ-ct in the *rad5Δ* and *mms2Δ* mutants than in the *rad18Δ* mutant thanks to its initial binding to ubiquitin on PCNA K164. It remains to be determined whether Pol η always requires PCNA mono-ubiquitination to efficiently outcompete Pol δ-ct or whether this requirement is less stringent for DNA lesions not induced by UV. Nevertheless, although PCNA undergoes mono-ubiquitination following UV radiation in the *rad5Δ* and *mms2Δ* mutants, WT Pol δ still competes with TLS polymerases. Therefore, the competition between Pol δ and the TLS polymerases could play a role in the choice leading to the template switching pathway. For instance, Pol δ preferential binding to PCNA in suitable genomic contexts could prevent TLS and allow PCNA poly-ubiquitination to occur more frequently. Conversely, genomic sequences that challenge the replication fork stability and the stable interaction between Pol δ and PCNA could favor the recruitment of TLS polymerases. Interestingly, no increase in UV resistance in absence of Rad51 is observed with Pol δ-ct (S1 and S7E Figs). This suggests that Pol δ regulator role in the presence of ubiquitinated PCNA might take place preferentially at stalled replication forks rather than at single-strand gaps behind the forks that could be processed by HR.

Finally, such a regulatory role for Pol δ in DNA damage tolerance could be also a key feature in TLS that occurs independently of PCNA ubiquitination (see Introduction). In agreement with this hypothesis, some genomic regions, such as repetitive sequence elements, hinder elongation by Pol δ and lead to its dissociation from the replication forks [78]. On the other hand, TLS polymerases are emerging as major actors in DNA synthesis at repetitive DNA sequences and their resulting non-B DNA structures [79]. Thus, a simple prediction would be that TLS polymerases sparsely rely on PCNA ubiquitination for their recruitment at intrinsically difficult to replicate loci. Other post-translational modifications might be at work in these situations, as exemplified by human Pol η recruitment to replication forks thanks to its sumoylation, but independently of PCNA ubiquitination [80].

## Materials and methods

### Yeast strains, plasmids and media

The *S*. *cerevisiae* strains used in the present study are isogenic derivatives of W303-1A [81] or FF18733 (*his7-2*, *leu2-3*,*112*, *lys1-1*, *trp1-289*, *ura3-52*) and are listed in S1 Table. Gene deletions were performed by using a PCR-mediated one-step replacement technique [82,83]. All deletions were confirmed by PCR amplification of genomic DNA. Meiotic segregation of the *pol30-K164R* allele was followed by PCR amplification with the primers P96 and P97. To study the effect of the *rev1-12* allele, *rev1Δ* strains were transformed with centromeric pBL820 plasmids carrying either *REV1* or *rev1-12* (a generous gift by Dr Peter Burgers) [29]. The genomic *pol3-R1043G* mutation was generated by using the plasmid pL11. pL11 is a derivative of pL6 obtained by cloning the C-terminal end of *POL3* (from +2503 of the ORF to 523 bp downstream the stop codon) in pRS306 [58]. The *pol3-R1043G* mutation was inserted in pL11 after site-directed mutagenesis performed in pL6. For targeting the mutation by the two-step transplacement procedure [84], pL11 was digested with *Hind*III (site located 425 bp downstream of the *pol3-R1043G* mutation). The *pol3-FI1002-1003AA* and *pol31-YF327-328AA* mutations were generated by using the pPOL519 and pPOL545 plasmids (kindly provided by Dr Louise Prakash), respectively [53]. For targeting the mutations, pPOL519 and pPOL545 were digested with *Kpn*I and *Mfe*I, respectively. The *pol32-FF344-345LL* mutations were generated using plasmid pL19 that was obtained by cloning the C-terminal end of *POL32* (from +273 of the ORF to 500 bp downstream the stop codon) in pRS306 of. Prior to transformation, pL19 was digested with *Xba*I.

All media were prepared as previously described [85]. Mutants were selected on YPD medium containing 300 mg/L of geneticin (Sigma) or nourseothricine (cloNAT; Werner BioAgents).

### UV irradiation and UV-induced mutagenesis

Cells in stationary phase were plated at appropriate dilutions on YPD and synthetic plates containing canavanine prior to UV irradiation. UV irradiation was performed using a 264nm source. Survival was determined as the number of cells forming colonies on YPD plates following exposure to a given UV dose divided by the number of cells forming colonies on YPD plates in absence of irradiation. Data used to draw graphs represent the mean ± SEM of at least 3 independent experiments. UV-induced mutagenesis was assessed with the *CAN1* forward-mutation assay. UV-induced mutagenesis frequencies were obtained by dividing the number of colonies growing on selective medium containing canavanine (*i*.*e*., canavanine-resistant, can^R^, cells) by the number of cells that survived irradiation. The number of can^R^ colonies obtained after irradiation was corrected by subtracting the number of can^R^ colonies present on the non-irradiated plates and corresponding to spontaneous mutation events. UV-induced mutagenesis in the *rad18Δ* background has been measured at 15 J/m^2^.

### Thermo- and HU-sensitivity

Yeast cells were directly picked from fresh YPD plates, suspended in sterile water, serially diluted and spotted onto plates. 5 μl of the various dilutions containing 625, 125, 25 and 5 cells were deposited for each sample. Replicates were made on YPD plates and YPD plates containing increasing concentrations of HU. For each experiment, one YPD plate without HU was incubated at 37°C. Plates were incubated for three days at 30°C.

### Synthetic lethality

Synthetic lethality between Pol δ mutant alleles was tested by using crosses, sporulation, tetrad dissection as well as genetic and PCR analyses. Meiotic segregation of a given allele was followed within tetrads by PCR analysis. Primers for *pol3-ct*: P13 (GCAGGAGAAAGTAGAACAATT) and P4187 (TACGCCTTCTTATGTAGCGC); Primers for *pol3-R1043G*: P217 (CATAAAGGCATTATACGATGTCG) and P4187; Primers for *pol31-D304N*: P180 (GATATTATGCCCGGAACCAATA) and P181 (TCAATCTTGACCGTCTCTGC); Primers for *pol3-FI1002-1003AA*: P182 (GGAGGCTTGATGAGCTTTATT) and P184 (CGGTCTCATTAGAATTGCAGC); Primers for *pol31-YF327-328AA*: P158 (AGTCCCTAGAATCAGCCGC) and P159 (GTCAATCTTGACCGTCTCTGC); Primers for *pol32-FF344-345LL*: P221 (GGAACATTGGAAAGCTTGTTG) and P222 (TGAGGGACAGAGAAGATTGG).

### Yeast two-hybrid assays

The strain CTY10-5d (*MATa*, *ade2-101*, *his3Δ200*, *leu2Δ1*, *trp1Δ901*, *gal4*, *gal80*, *URA3*::*lexAop-lacZ*) was used for two-hybrid analysis. Pol31 was fused in frame with the lexA binding domain in plasmid pBTM116, while the Pol3 C-terminal amino-acids 1032–1097 were fused with the Gal4 activating domain of pACT2 to generate the plasmid pACT2-Pol3-ZnF2 [74]. The Pol3-R1043G, Pol31-YF327-328AA and Pol31-D304N mutations were introduced by site-directed mutagenesis and were confirmed by DNA sequencing. The resulting plasmids were called pACT2-Pol3-R1043G, pBTM116-Pol31-YF and pBTM116-Pol31-D304N, respectively. CTY10-5d was co-transformed with the pACT2 and pBTM116 plasmids that carry WT or mutated Pol3 CTD or Pol31 (Fig 5B). Cells were plated in -Leu -Trp selective medium supplemented with methionine at 22°C or 30°C for 3 to 4 days, and tested for β-galactosidase production in an overlay plate assay [59].

### Isothermal Titration Calorimetry (ITC)

Recombinant *S*. *cerevisiae* PCNA with an His-Tag was produced in *E coli* using the pET28c bacterial expression vector (Novagen) as described in [4] and purified on a 5 ml HisTrap column (GE Healthcare). The synthetic peptides containing the PIP-box motifs of *S*. *cerevisiae* Pol3, Pol31, Pol32 and Msh6 were purchased from Genecust at 95% purity, and the concentrations of the stock peptide solutions were determined by amino acid composition.The interactions between PCNA and the different PIP-box peptides were determined by isothermal titration calorimetry (ITC) using a VP-ITC calorimeter (Microcal). Prior to measurements, all solutions were degassed under vacuum. The ITC reaction cell (vol 1.8 mL) was loaded with 20–25 μM of PCNA solution. The syringe (500 μL) was filled with the different PIP-box peptides at a concentration of 270 μM. Proteins were extensively dialyzed against buffer T (20 mM Tris [pH 7.5], 50 mM NaCl, and 20 mM β-mercaptoethanol) before ITC. The thermodynamic parameters ΔH, N, and Ka were obtained by non-linear least-squares fitting of the experimental data using the single set of independent binding sites model of the Origin software provided with the instrument. The free energy of binding (ΔG) and the entropy (ΔS) were determined using the classical thermodynamic formula, ΔG = -RT ln(Ka) and ΔG = ΔH -TΔS. All binding experiments were performed in duplicate or triplicate at 30°C.

## Supporting information

S1 Fig *pol3-ct* effect on UV resistance of yeast cells deficient for HR or the error-prone DDT pathway. Survival curves of haploid cells after exposure to UV light. (A) *pol3-ct* does not affect the UV sensitivity of *rad51Δ* cells. (B and C) *pol3-ct* marginally affects *rev3Δ* and *rev1Δ* cells UV sensitivity.
(EPS)

S2 Fig *pol3-ct* increases UV resistance of PCNA polyubiquitination-deficient cells through Pol ζ and Pol η activation. Survival curves of haploid cells after exposure to UV light. (A) *pol3-ct* partially suppresses UV sensitivity associated with *rad5Δ*. (B) *pol3-ct* does not suppress UV sensitivity of the *rad5Δ rad30Δ* double mutant. (C) *pol3-ct* does not suppress UV sensitivity of the *rad5Δ rev3Δ* double mutant.
(EPS)

S3 Fig HU- and thermo-sensitivities of structural Pol δ mutants. (A) Single mutants and double mutants with *pol3-ct*. (B) Different genetic combinations between the *pol3-FI*, *pol31YF* and *pol32-pip* alleles. Note that the triple mutant in the W303 background is viable and not thermosensitive.
(EPS)

S4 Fig Synthetic lethality between mutant alleles of *POL3*, *POL31* and *POL32*. (A) Following sporulation of diploid cells, four-spore asci were dissected on YPD plates. Spores individually separated under the microscope formed colonies after incubation at 30°C for three days. Images of 12 dissected asci are shown. The relevant genotype of the haploid parental strains for each cross (symbolized by x) is indicated underneath the image of the exemplified dissection plate. Genetic and PCR analyses allowed following the meiotic segregation of each allele of interest in each tetrad (see Materials and Methods). Circles highlight spores that could not form a colony because they carry the two tested mutant alleles, thereby demonstrating the synthetic lethality between these alleles. The two alleles showed as examples are *pol3-R1043G* and *pol32Δ*. (B) Summary of the synthetic lethality found between mutant alleles of the genes that encode the three Pol δ subunits Pol3, Pol31 and Pol32. *POL3* mutant alleles are shown in blue. *POL31* mutant alleles are in red. *POL32* mutant alleles are in green. SL: synthetic lethality between the tested alleles; Viable: spores carrying the two mutant alleles can grow. Note that *pol3-ct* and *pol3-FI* display similar phenotypes. Moreover, while *pol32Δ* shows synthetic lethality with all tested alleles, *pol32-pip* is viable. Finally, spores carrying *pol3-FI*, *pol31-YF* and *pol32-pip* simultaneously can form colonies.
(EPS)

S5 Fig UV-induced mutagenesis in Pol δ structural mutants. Each dot represents the can^R^ frequency obtained for one experiment for each strain. The median value for each strain is represented by a horizontal bar: ***, *P* = 0.0001 (Mann-Whitney test between WT and *pol31-D304N*).
(EPS)

S6 Fig The proposed Pol3 and Pol31 PIP motifs are not conserved. Multiple sequence alignments of the potential motifs found in Pol3, Pol31 and Pol32 show the conservation of the Pol32 PIP residues and the lack of conservation for the sequences of the Pol3 and Pol31 motifs. The potential position of the canonical PIP-box residues is indicated by a star. Conservation of the residue at the PIP position is underlined in yellow. Color code: green for hydrophobic residues (V,L,I,M); light green for tryptophan (W); cyan for aromatic (F,Y); blue for basic residues (R,K); red for acidic residues (D,E); grey blue for polar (S,T,Q,N); brown for cysteine (C); orange for glycine (G); light blue for Alanine (A); light brown for proline (P); magenta for histidine (H).
(EPS)

S7 Fig Summary figure: Impact of Pol δ-ct on the choice of DDT pathway in the presence of PCNA ubiquitination. (A) Schematic shows a stalled 3’end primer terminus at a pyrimidine dimer with PCNA ubiquitinatination (red circles) as a consequence of the stalling. Pol δ-ct residence time at the junction and on PCNA can be shorter than that of WT Pol δ. (B) The *rad30Δ* mutant is UV sensitive and *pol3-ct* partially suppresses this sensitivity. This suppression is no longer observed in the *rad51Δ* mutant, thereby suggesting that in this case, UV-induced DNA lesions can be channeled towards the HR pathway more frequently thanks to Pol3-ct (red dashed arrow). (C) In the *rev3Δ* and *rev1Δ* mutants, the error-prone pathway is abolished and the mutants display UV sensitivity. *pol3-ct* has a minor effect in these mutants and might weakly favor Pol η recruitment (dashed red arrow). (D) In the *rad5Δ* and *mms2Δ* mutants, the template switching (TS) pathway is defective. *pol3-ct* suppresses UV sensitivity associated with *rad5Δ* and *mms2Δ*, and this effect depends on Pol η and Pol ζ. Thus, UV DNA lesions are channeled to the TLS pathways in the Pol δ-ct mutant (red arrows). (E) In the *rad51Δ* mutant, *pol3-ct* has no effect upon UV radiation, thereby indicating no additional channeling of UV-induced DNA lesions from the HR pathway to the other DDT pathways.
(EPS)

S1 Table Yeast strains used in this work. (XLS)
