# Correction: The translesion DNA polymerases Pol ζ and Rev1 are activated independently of PCNA ubiquitination upon UV radiation in mutants of DNA polymerase δ

**DOI:** 10.1371/journal.pgen.1007236

**Published:** 2018-02-14

**Authors:** Carine Tellier-Lebegue, Eléa Dizet, Emilie Ma, Xavier Veaute, Eric Coïc, Jean-Baptiste Charbonnier, Laurent Maloisel

S5 Fig is a duplicate of Fig 5. Please view the correct S5 Fig below. The publisher apologizes for the error.

## Supporting information

S5 Fig UV-induced mutagenesis in Pol δ structural mutants. Each dot represents the can^R^ frequency obtained for one experiment for each strain. The median value for each strain is represented by a horizontal bar: ***, *P* = 0.0001 (Mann-Whitney test between WT and *pol31-D304N*).
(EPS)
